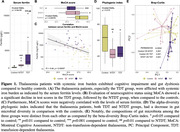# Regularity of blood transfusion influences the severity of systemic iron burden, cognitive decline, and gut dysbiosis in thalassemia patients

**DOI:** 10.1002/alz.085169

**Published:** 2025-01-03

**Authors:** Kanokphong Suparan, Kornkanok Trirattanapa, Sirawit Sriwichaiin, Sasiwan Kerdphoo, Adisak Tantiworawit, Nipon Chattipakorn, Siriporn C Chattipakorn

**Affiliations:** ^1^ Immunology Unit, Department of Microbiology, Faculty of Medicine, Chiang Mai University, Chiang Mai Thailand; ^2^ Center of Excellence in Cardiac Electrophysiology Research, Chiang Mai University, Chiang Mai Thailand; ^3^ Neurophysiology Unit, Cardiac Electrophysiology Research and Training Center, Faculty of Medicine, Chiang Mai University, Chiang Mai Thailand; ^4^ Division of Hematology, Department of Internal Medicine, Faculty of Medicine, Chiang Mai University, Chiang Mai Thailand; ^5^ Cardiac Electrophysiology Unit, Department of Physiology, Faculty of Medicine, Chiang Mai University, Chiang Mai Thailand; ^6^ Department of Oral Biology and Diagnostic Sciences, Faculty of Dentistry, Chiang Mai University, Chiang Mai Thailand

## Abstract

**Background:**

Thalassemia is a hereditary disease with impaired red blood cell production, resulting in cumulative systemic iron burden. The life‐long therapeutic blood transfusion with or without iron chelators in those patients leads to the development of early‐onset neurocognitive decline. However, the effects of regularity of blood transfusion on the severity of iron burden, cognitive decline, and gut dysbiosis in thalassemia patients are still unclear.

**Method:**

Sixty participants, including 20 transfusion‐dependent (TDT) patients, 20 non‐transfusion‐dependent (NTDT) patients, and 20 healthy controls, underwent evaluation for neurocognitive function using the Montreal Cognitive Assessment (MoCA). The current systemic iron burden was determined by the levels of serum ferritin. Amplicon‐based sequencing of the bacterial 16s rRNA V3‐V4 regions of stool DNA was employed to delineate profiles of gut microbiota.

**Result:**

The TDT patients showed greater severity of systemic iron burden, cognitive decline and gut dysbiosis than the NTDT patients. The TDT group was particularly afflicted with systemic iron burden as indicated by the highest levels of serum ferritin (**Fig. 1A**). Compared to the controls, the TDT and NTDT groups had significantly lower MoCA scores (**Fig. 1B**). In addition, there was a negative correlation between MoCA scores and the levels of serum ferritin (**Fig. 1C**). The thalassemia patients also exhibited gut dysbiosis, with microbial compositions among the three groups being distinct from each other (**Fig. 1D** and **1E**). Interestingly, an increase in abundance of *Paraclostridium* was associated with cognitive decline.

**Conclusion:**

These findings suggest that the severity of systemic iron burden, based on the regularity of blood transfusion, impacts on the magnitude of cognitive decline and gut dysbiosis in thalassemia patients. It is possible that systemic iron potentially serves as a dose‐dependent factor in the pathogenesis of neurodegeneration through gut dysbiosis. Therefore, therapeutic approaches via well‐control of systemic iron levels and balanced gut microbiota could prevent early‐onset cognitive decline in thalassemia patients.